# A Phase 1 Dose-Escalation Study of PF-06671008, a Bispecific T-Cell-Engaging Therapy Targeting P-Cadherin in Patients With Advanced Solid Tumors

**DOI:** 10.3389/fimmu.2022.845417

**Published:** 2022-04-14

**Authors:** James J. Harding, Ignacio Garrido-Laguna, Xiaoying Chen, Cynthia Basu, Afshin Dowlati, Alison Forgie, Andrea T. Hooper, Cris Kamperschroer, Steven I. Max, Allison Moreau, Megan Shannon, Gilbert Y. Wong, David S. Hong

**Affiliations:** ^1^ Memorial Sloan Kettering Cancer Center and Weill Cornell Medical College, New York, NY, United States; ^2^ Huntsman Cancer Institute at University of Utah, Salt Lake City, UT, United States; ^3^ Early Oncology Development and Clinical Research, Worldwide Research and Development, Pfizer, San Diego, CA, United States; ^4^ University Hospitals Seidman Cancer Center and Case Western Reserve University, Cleveland, OH, United States; ^5^ Early Clinical Development and Oncology Research, Worldwide Research and Development, Pfizer, San Francisco, CA, United States; ^6^ Oncology Research and Development, Pfizer, Inc., Pearl River, NY, United States; ^7^ Drug Safety Research and Development, Worldwide Research and Development, Pfizer, Groton, CT, United States; ^8^ Janssen Pharmaceutical Companies of Johnson & Johnson, Philadelphia, PA, United States; ^9^ Department of Investigational Cancer Therapeutics, The University of Texas MD Anderson Cancer Center, Houston, TX, United States

**Keywords:** P-cadherin, solid tumor, T-cell–redirecting bispecific antibody, immunotherapy, phase 1, cytokine release syndrome (CRS)

## Abstract

P-cadherin is a cell-cell adhesion molecule that is overexpressed in several solid tumors. PF-06671008 is a T-cell–redirecting bispecific antibody that engages both P-cadherin on tumors and CD3ϵ on T cells and induces antitumor activity in preclinical models. We conducted a phase 1, open-label, first-in-human, dose-escalation study to characterize the safety and tolerability of PF-06671008, towards determining the recommended phase 2 dose. Adult patients with treatment-refractory solid tumors received PF-06671008 (1.5–400 ng/kg) as a weekly intravenous (IV) infusion on a 21-day/3-week cycle. Parallel cohorts evaluated dosing *via* subcutaneous injection (SC) or an IV-prime dose. Of the 27 patients enrolled in the study, 24 received PF-06671008 IV in escalating doses, two received SC, and one IV-prime. A dose-limiting toxicity of cytokine release syndrome (CRS) occurred in the 400-ng/kg IV group, prompting evaluation of SC and IV-prime schedules. In all, 25/27 patients who received PF-06671008 reported at least one treatment-related adverse event (TRAE); the most common were CRS (21/27), decreased lymphocyte count (9/27), and hypophosphatemia (8/27). Seven patients permanently discontinued treatment due to adverse events and no treatment-related deaths occurred. Cytokine peak concentrations and CRS grade appeared to positively correlate with C_max_. Although the study was terminated due to limited antitumor activity, it provides important insights into understanding and managing immune-related adverse events resulting from this class of molecules.

**Clinical Trial Registration:**

URL: https://clinicaltrials.gov/ct2/show/NCT02659631, ClinicalTrials.gov Identifier: NCT02659631.

## Introduction

Bispecific T-cell–redirecting antibodies bind to both tumor-associated antigens (TAA) and the signaling components of the T-cell receptor complex, bypassing the normal T-cell receptor–major histocompatibility complex interaction, thus enabling the cytotoxic activity of T cells to be redirected toward the recognized tumor cells. The effectiveness of bispecific T-cell–redirecting antibodies is exemplified by blinatumomab, a bispecific T-cell engager (BiTE) targeting CD19 and CD3 that improves the overall survival of patients with relapsed/refractory B-cell–precursor acute lymphoblastic leukemia (1). The efficacy of blinatumomab has sparked intense interest in the application of bispecific T-cell–redirecting antibodies for the durable treatment of not only hematologic malignancies (2) but also solid tumors (3, 4).

The classical type 1 cadherins, which include E-cadherin (*CDH1*), N-cadherin (*CDH2*), and P-cadherin (*CDH3*), are a family of calcium-dependent, cell-cell adhesion molecules (5, 6). Although their intracellular domains have no enzymatic activity, cadherins, through binding various partners and co-factors, play a critical role in signal transduction that ultimately influences several important biological processes, such as tissue development, cell migration, and tumorigenesis (7). P-cadherin is overexpressed relative to normal tissue in a variety of invasive solid tumors (e.g., gastric, pancreatic, colon, prostate, endometrial, and non-small cell lung cancers) and represents a potential target for novel therapies, including T-cell engagers (8, 9). Preclinical models indicate that interference with tumoral P-cadherin impairs cancer migration and suppresses cancer growth (10, 11). Importantly, P-cadherin has recently been identified as a novel TAA in digestive cancers, and P-cadherin–specific cytotoxic lymphocytes effectively inhibit the growth of human cancer in *in vitro* and *in vivo* models (10).

Given these preclinical findings, PF-06671008, a T-cell–redirecting bispecific molecule that targets both P-cadherin and cluster of differentiation 3 epsilon (CD3ϵ) on T cells was developed using the Dual Affinity Re-Targeting (DART) platform (12). DART molecules are similar in principle to BiTE molecules but are engineered to address known limitations with existing bispecific engineering technologies, namely reduced binding potency due to steric constraints and molecular instability (12). PF-06671008 consists of an Fc fusion to a diabody, with one binding domain targeting P-cadherin and the other targeting CD3ϵ in the T-cell receptor complex (13). The variable fragments in DART proteins are comprised of a VL partner on one polypeptide chain and a VH partner on a separate chain. The DART molecule was further engineered to extend its half-life through fusion to the Fc domain of human immunoglobulin; without extension of its half-life, therapy with the molecule would likely require administration *via* continuous infusion. Mutations were also introduced into the Fc domain to encourage heterodimerization and to prevent binding to FcγR and effector function (13).

*In vitro* studies demonstrated that engagement of P-cadherin and CD3 by PF-06671008 resulted in dose-dependent T-cell activation and cytokine release, as well as target cell lysis (14). Likewise, PF-06671008 administration resulted in dose-dependent tumor regression in mice with established tumor xenografts; tumor regression was observed for both cell line- and patient-derived xenografts of human triple-negative breast cancer and colorectal cancer (14). The degree of PF-06671008–induced tumor regression correlated with the level of P-cadherin expression in tumor cells. Importantly, preclinical data showed that cytokine release syndrome (CRS), a known class effect of T-cell engagers, could be modulated by both dosing schedule and route of administration. Reduced cytokine levels were previously observed in animal studies for intravenous (IV) priming (IV-prime) with an initial lower dose and for subcutaneous (SC) dosing, as compared with IV administration (15, 16).

Based on the promising antitumor activity observed in preclinical models, a phase 1 dose-escalation study of single-agent PF-06671008 was initiated in adult patients with advanced solid tumors associated with elevated P-cadherin expression. Here, we report on the safety, pharmacokinetic (PK), immunogenicity, and pharmacodynamic characteristics and preliminary antitumor activity of PF-06671008 with IV and SC administration in the dose-escalation portion of this first-in-human clinical trial.

## Methods

### Study Design and Treatment

This study was a phase 1, open-label, multicenter, dose-escalation, first-in-human study of single-agent PF-06671008 (ClinicalTrials.gov, NCT02659631). The study design consisted of a dose-escalation portion (part 1) followed by a dose-expansion portion (part 2). The primary objective of part 1 was to evaluate the safety and tolerability of PF-06671008 in order to determine the maximum tolerated dose (MTD) and the recommended phase 2 dose (RP2D). The secondary objectives were to characterize the overall safety profile, PK, immunogenicity, and antitumor activity of PF-06671008. Exploratory objectives included characterization of cytokines and chemokines.

In part 1 of the study, sequential cohorts of patients with tumor types with potential P-cadherin expression received escalating doses of PF-06671008. Weekly IV and SC routes of administration were explored, including an IV-priming strategy, in which an initial single, lower dose (next immediately lower dose previously shown to be tolerated) was administered, followed by a higher maintenance dose. Expansion cohorts at the RP2D were planned, but not completed, for patients with P-cadherin expressing triple-negative breast cancer, colorectal cancer, and non-small cell lung cancer in part 2 of the study.

This study followed the Declaration of Helsinki and International Conference on Harmonization Good Clinical Practice guidelines and was approved by the institutional review boards of the following institutions: MD Anderson Cancer Center, The University of Utah, Memorial Sloan Kettering Cancer Center, and University Hospitals. All patients provided written informed consent prior to study commencement.

### Patients

Adult patients (aged ≥18 years) were eligible for enrollment if they had a histological or cytological diagnosis of a tumor type with potential P-cadherin expression and were refractory to or intolerant of established treatments known to provide clinical benefit. Tumor types were chosen based on transcript and/or protein expression data from previously published literature and included bladder urothelial carcinoma, breast carcinoma, colorectal adenocarcinoma, head and neck squamous cell carcinoma, non-small cell lung cancer, ovarian (epithelial) carcinoma, pancreatic adenocarcinoma, and prostate adenocarcinoma. Patients had Eastern Cooperative Oncology Group performance status ≤1 as well as adequate bone marrow, renal, and liver function. Key exclusion criteria were central nervous system disease; a history of or active seizure or autoimmune disorders; clinically significant bacterial, fungal, or viral infections; and radiation therapy within 4 weeks before registration or systemic anticancer therapy within 4 weeks of registration (6 weeks for mitomycin C or nitrosoureas).

### Treatment

**Figure 1:**
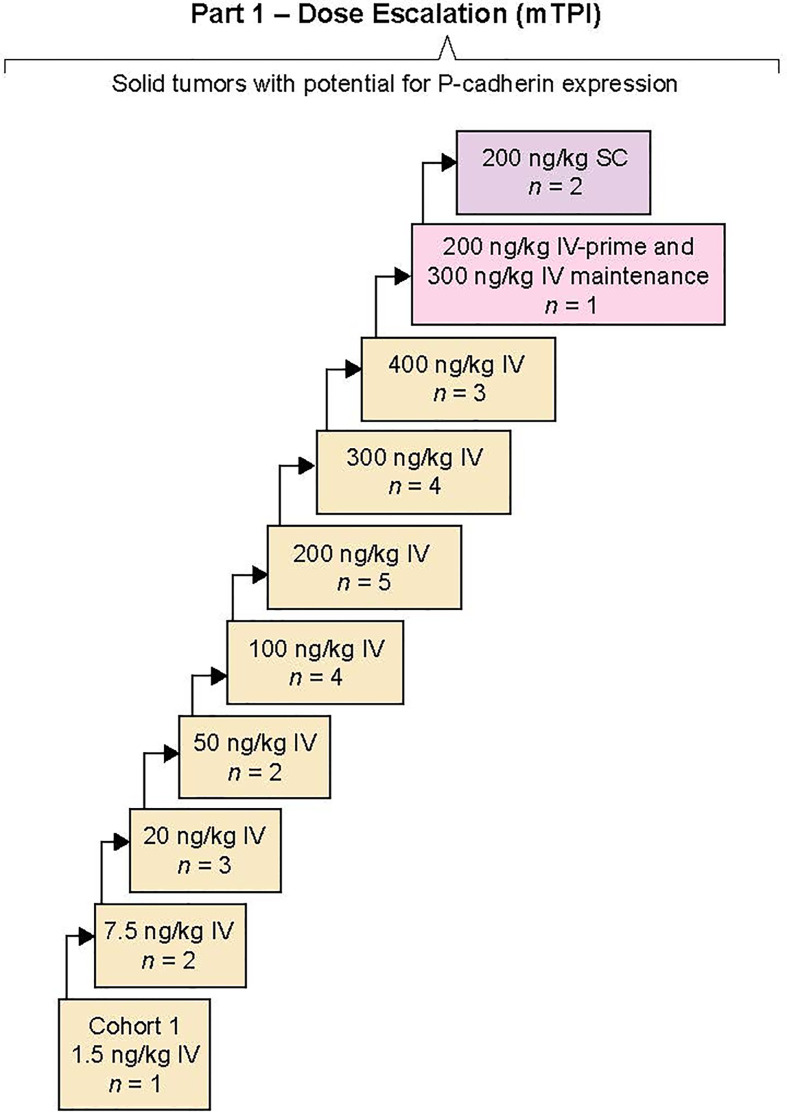
Study design. A first-in-human, open-label, multicenter, phase 1 study of PF-00671008 in advanced solid tumors. The primary objective of part 1 of the study was to determine the maximum tolerated dose and the recommended phase 2 dose. Sequential dose escalation was completed by a modified toxicity probability interval design evaluating intravenous (IV) and subcutaneous (SC) routes. Part 2 was not completed.

Patients received PF-06671008 as a weekly IV infusion or SC injection; one cycle was 21 days or 3 weekly treatments, and dose escalation was initiated with a minimal anticipated biological effect level (MABEL)-derived starting dose of 1.5 ng/kg for the first cohort (**Figure 1**). Prior clinical data with blinatumomab (a BiTE) (17) and our preclinical findings suggested that a lower initial “priming” dose (IV-prime) might attenuate CRS. Our preclinical findings further suggested that SC dosing may also reduce CRS. Therefore, an IV-priming strategy and a parallel SC-dosing strategy were included as part of the protocol. At dose levels where CRS grade ≥3 lasting for >24 hours occurred in more than two patients in a cohort, or CRS grade ≥4 occurred in one or more patients in a cohort, a parallel cohort was opened to evaluate an IV-prime dose or a SC dose. The IV-prime dose consisted of the next lowest dose that had been previously determined to be tolerated, which would then be administered as the first dose in the newly opened cohort. This would be followed by a higher maintenance dose in the same cohort. Using this scheme, doses could be escalated in subsequent cohorts according to dose-escalation rules. In other words, on cycle 1 day 1, patients received a previously cleared lower dose of PF-06671008 and, if tolerable, additional doses in the cycle occurred at the maintenance dose per the dose-escalation scheme. Patients continued treatment until progression of disease, intolerable toxicity, or withdrawal of consent.

### Study Assessments

#### Safety

Safety evaluations included medical history, physical examinations, and clinical laboratory measurements. On cycle 1 day 1 of standard dosing and on both cycle 1 days 1 and 8 of IV-prime dosing, patients were admitted for continuous safety monitoring for at least 24 (IV dosing) or 48 (SC dosing) hours. Adverse events (AEs) were monitored and graded by National Cancer Institute Common Terminology Criteria for Adverse Events v.4.03.

#### Dose-Limiting Toxicities (DLT) Definition

The following AEs were considered DLTs if they occurred in the first treatment cycle (21 days after the first dose), unless there was a clear alternative explanation: grade 4 neutropenia; grade ≥3 febrile neutropenia; grade ≥3 neutropenia with infection; grade 3 thrombocytopenia with significant bleeding; grade 4 thrombocytopenia; grade ≥3 toxicities (not CRS) that were considered clinically significant; grade ≥3 CRS maximally treated that last >72 hours; and any persistent treatment-related toxicities that delayed administration of the next scheduled dose by >2 weeks.

#### Pharmacokinetics and Immunogenicity

For patients receiving IV administration, blood samples for PK analysis were collected at days 1, 2, 4, 8, 9, 11, and 15 of cycle 1; days 1, 2, 4, 8, and 15 of cycle 2; days 1, 8, and 15 of subsequent cycles; and at the end of treatment. For patients receiving SC administration, blood samples for PK analysis were collected at the following protocol-defined time points: days 1, 2, 3, 5, 8, 9, 10, and 15 of cycle 1; days 1, 2, 3, 8, and 15 of cycle 2; days 1, 8, and 15 of subsequent cycles; and at end of treatment.

The serum concentration of PF-06671008 was quantified using a validated electrochemiluminescence method and non-compartmental analysis was used to estimate standard PK parameters, which included maximum serum concentration (C_max_), time to maximum serum concentration (T_max_), area under the serum concentration versus time profile from time zero to tau (tau=168 hr) (AUC_tau_), and if data permitted, terminal half-life (t_1/2_). For all patients, blood samples for the analysis of antidrug antibodies (ADAs) against PF-06671008 were collected on days 1 and 15 of cycle 1, on day 1 of subsequent cycles, and at end of treatment.

#### Biomarker and Pharmacodynamic Assessments

Blood samples for quantification of serum cytokines and chemokines were collected at defined time points. Cytokines were quantified using a Luminex bead-based assay. The assay was used to simultaneously detect 12 cytokines (interleukin [IL]-1β, IL-2, IL-4, IL-5, IL-6, IL-8, IL-10, IL-12, IL-13, IL-17; tumor necrosis factor [TNF]-α; interferon [IFN]-γ); and the soluble IL-2 receptor alpha subunit (CD25). Briefly, serum samples were diluted and incubated with a set of beads attached to different anti-cytokine monoclonal antibodies. Samples were then incubated with a biotinylated secondary polyclonal antibody before a final incubation with streptavidin conjugated R-phycoerythrin, which served as the reporter. The intensity of R-phycoerythrin fluorescence for each bead was quantified using a Luminex analyzer.

#### Antitumor Activity

Assessments of tumor response were performed using computed tomography (chest, abdomen, and pelvis) or magnetic resonance imaging scans every 6 weeks from cycle 3 until disease progression, death, or permanent discontinuation of the study treatment; from cycle 8, scans were conducted every 12 weeks. Complete and partial responses had to be confirmed by a repeat assessment at least 4 weeks after the initial response, per Response Evaluation Criteria in Solid Tumors (RECIST) v1.1 (18).

#### Statistical Analyses

The safety analysis set consisted of all enrolled patients who received at least one dose of study treatment. This study was designed to establish the MTD, which was defined as the dose that yields an ~27.5% probability of DLT and considers equivalent doses that yield a probability of DLT in the (equivalence) interval between 22.5 and 32.5%. A Bayesian method to guide dose escalation with modified toxicity probability interval design used in this study computes the posterior probability of three dosing intervals reflecting the relative differences between the rate of toxicity of each dose level to the target rate (pT = 0.275).

Part 1 of the study was expected to enroll a maximum of 92 patients. The full analysis set consisted of all enrolled patients and the safety analysis set included all enrolled patients who received at least one dose of study treatment. All patients treated with one or more doses of study treatment were also included in the PK analyses for both concentration and parameters.

## Results

### Patients and Treatment

A total of 27 patients received PF-06671008 across 10 dose-escalation and dose-regimen groups. Initially, 24 patients received PF-06671008 IV in single-agent dose escalation, with doses ranging from 1.5 ng/kg to 400 ng/kg. The median number of treatment cycles started was 2 (range, 1–16). Most patients were White (*n*=21/27) and more than half of the patients were male (*n*=15/27). Patients ranged in age from 26 to 75 years, with a mean age of 55.9 years. The most common tumor types among the patients enrolled (*N*=27) were colorectal cancer (*n*=11/27), followed by breast (*n*=5/27), prostate (*n*=4/27), and pancreatic (*n*=4/27) cancer (**Table 1**). Most patients (*n*=16/27) had received more than three lines of prior systemic therapy (**Table 1**).

**Table 1:** Patient demographics and baseline characteristics.

	PF-06671008 dose, ng/kg											
	1.5 IV (*n* = 1)	7.5 IV (*n* = 2)	20 IV (*n* = 3)	50 IV (*n* = 2)	100 IV (*n* = 4)	200 IV (*n* = 5)	300 IV (*n* = 4)	400 IV (*n* = 3)	200 IV prime and 300 IV maintenance (*n* = 1)	200 SC (*n* = 2)	Total (*n* = 27)
Sex, *n*											
Male	1	1	1	0	3	1	4	2	1	1	15
Female	0	1	2	2	1	4	0	1	0	1	12
Race, *n*											
White	1	1	3	2	3	3	3	2	1	2	21
Black	0	1	0	0	1	2	0	0	0	0	4
Asian	0	0	0	0	0	0	0	1	0	0	1
Other	0	0	0	0	0	0	1	0	0	0	1
Age, mean (range), y	63.0 (63–63)	55.0 (49–61)	50.0 (43–55)	51.5 (37–66)	66.3 (65-67)	53.0 (26–75)	61.5 (44–72)	59.3 (46–71)	58.0 (58–58)	36.0 (34–38)	55.9 (26–75)
ECOG PS, *n* (%)											
0	0	1 (50.0)	2 (66.7)	0	2 (50.0)	2 (40.0)	1 (25.0)	1 (33.3)	0	1 (50.0)	10 (37.0)
1	1 (100.0)	1 (50.0)	1 (33.3)	2 (100)	2 (50.0)	3 (60.0)	3 (75.0)	2 (66.7)	1 (100.0)	1 (50.0)	17 (63.0)
Primary diagnosis											
Colorectal cancer	1	1	2	–	–	2	2	1	1	1	11
Breast cancer	–	–	–	1	1	2	–	–	–	1	5
Prostate cancer	–	–	–	–	1	–	2	1	–	–	4
Pancreatic cancer	–	1	–	1	2	–	–	–	–	–	4
Ovarian cancer	–	–	–	–	–	1	–	–	–	–	1
Head and neck cancer	–	–	1	–	–	–	–	1	–	–	2
Number of prior systemic therapies, *n* (%)											
1	0	0	0	0	0	0	0	0	0	0	0
2	0	0	0	0	1 (25.0)	1 (20.0)	0	0	0	0	2 (7.4)
3	1 (100.0)	1 (50.0)	2 (66.7)	1 (50.0)	1 (25.0)	2 (40.0)	0	1 (33.3)	0	0	9 (33.3)
>3	0	1 (50.0)	1 (33.3)	1 (50.0)	2 (50.0)	2 (40.0)	4 (100.0)	2 (66.7)	1 (100.0)	2 (100.0)	16 (59.3)

ECOG PS, Eastern Cooperative Oncology Group performance status; IV, intravenous; SC, subcutaneous.

### Safety

All 27 patients had at least one all-causality AE and 25 patients had at least one treatment-related AE (TRAE). The most common (*n*=21/27) TRAE was CRS (**Table 2**). Five patients discontinued the study treatment due to CRS. CRS resulted in escalation of care to the intensive care unit for 7/27 (25.9%) patients and 4/27 (14.8%) patients with CRS required vasopressors; none required intubation. In total, 2/27 (7.4%) and 6/27 (22.2%) patients received corticosteroids and tocilizumab for the treatment of CRS, respectively. Other commonly reported TRAEs were decreased lymphocyte count (*n*=9/27), hypophosphatemia (*n*=8/27), nausea (*n*=8/27), and fatigue (*n*=7/27) (**Table 2**).

**Table 2:** Treatment-related adverse events reported in ≥5% of patients (*N* = 27)^a^.

Number of events (%)	Grade 1	Grade 2	Grade 3	Grade 4	Grade 5	Total
Any adverse event	2 (7.4)	6 (22.2)	10 (37.0)	7 (25.9)	0	25 (92.6)
Cytokine release syndrome	4 (14.8)	12 (44.4)	5 (18.5)	0	0	21 (77.8)
Lymphocyte count decreased	0	0	2 (7.4)	7 (25.9)	0	9 (33.3)
Hypophosphatemia	1 (3.7)	2 (7.4)	4 (14.8)	1 (3.7)	0	8 (29.6)
Nausea	5 (18.5)	3 (11.1)	0	0	0	8 (29.6)
Fatigue	3 (11.1)	3 (11.1)	1 (3.7)	0	0	7 (25.9)
Diarrhea	3 (11.1)	0	2 (7.4)	0	0	5 (18.5)
Pyrexia	3 (11.1)	1 (3.7)	1 (3.7)	0	0	5 (18.5)
Arthralgia	2 (7.4)	2 (7.4)	0	0	0	4 (14.8)
Chills	3 (11.1)	1 (3.7)	0	0	0	4 (14.8)
Vomiting	1 (3.7)	3 (11.1)	0	0	0	4 (14.8)
Constipation	3 (11.1)	0	0	0	0	3 (11.1)
Pruritus	2 (7.4)	0	1 (3.7)	0	0	3 (11.1)
White blood cell count decreased	2 (7.4)	0	1 (3.7)	0	0	3 (11.1)
Decreased appetite	1 (3.7)	1 (3.7)	0	0	0	2 (7.4)
Dizziness	2 (7.4)	0	0	0	0	2 (7.4)
Dyspnea	0	0	2 (7.4)	0	0	2 (7.4)
Electrocardiogram QT prolonged	1 (3.7)	1 (3.7)	0	0	0	2 (7.4)
Gastroesophageal reflux disease	0	2 (7.4)	0	0	0	2 (7.4)
Headache	2 (7.4)	0	0	0	0	2 (7.4)
Hypertension	0	1 (3.7)	1 (3.7)	0	0	2 (7.4)
Hypocalcemia	1 (3.7)	1 (3.7)	0	0	0	2 (7.4)
Hypoxia	0	2 (7.4)	0	0	0	2 (7.4)
Platelet count decreased	1 (3.7)	1 (3.7)	0	0	0	2 (7.4)
Tachycardia	1 (3.7)	1 (3.7)	0	0	0	2 (7.4)

a A patient was counted only once at the highest grade of the corresponding adverse event.

During the IV-dose escalation, one patient in the 400 ng/kg IV group had CRS, which was considered a DLT (grade 3 CRS maximally treated lasting >72 hours). This DLT led to the evaluation of an IV-prime dose, with one patient enrolled in the IV-prime dose group at 200 ng/kg, followed by 300 ng/kg IV for maintenance. Additionally, following identification of 200 ng/kg IV as the IV-prime dose, two patients were enrolled in the 200 ng/kg SC cohort.

A total of seven of 27 patients discontinued from the study due to AEs and nine patients experienced AEs that resulted in temporary discontinuation or dose reductions. In the 200 ng/kg IV group, four of five patients had serious AEs (SAEs) and all patients had at least one grade 3 or 4 AE, but none that met DLT criteria. In this group, two of five patients had dose reductions due to AEs and three of five had treatment temporarily discontinued due to AEs. Of the two patients in the 200 ng/kg SC group, neither had SAEs, grade 3 or 4 AEs, or AEs that resulted in dose reductions or temporary discontinuation of treatment. The patient who received a 200 ng/kg IV-priming dose followed by 300 ng/kg IV for maintenance had no SAEs and no AEs leading to dose reductions or temporary discontinuation of treatment.

During the treatment period, there were two deaths due to disease and 14 deaths occurred during the follow-up period. None of the deaths were directly related to PF-06671008 treatment. The MTD was not determined.

### Pharmacokinetics and Immunogenicity

**Figure 2:**
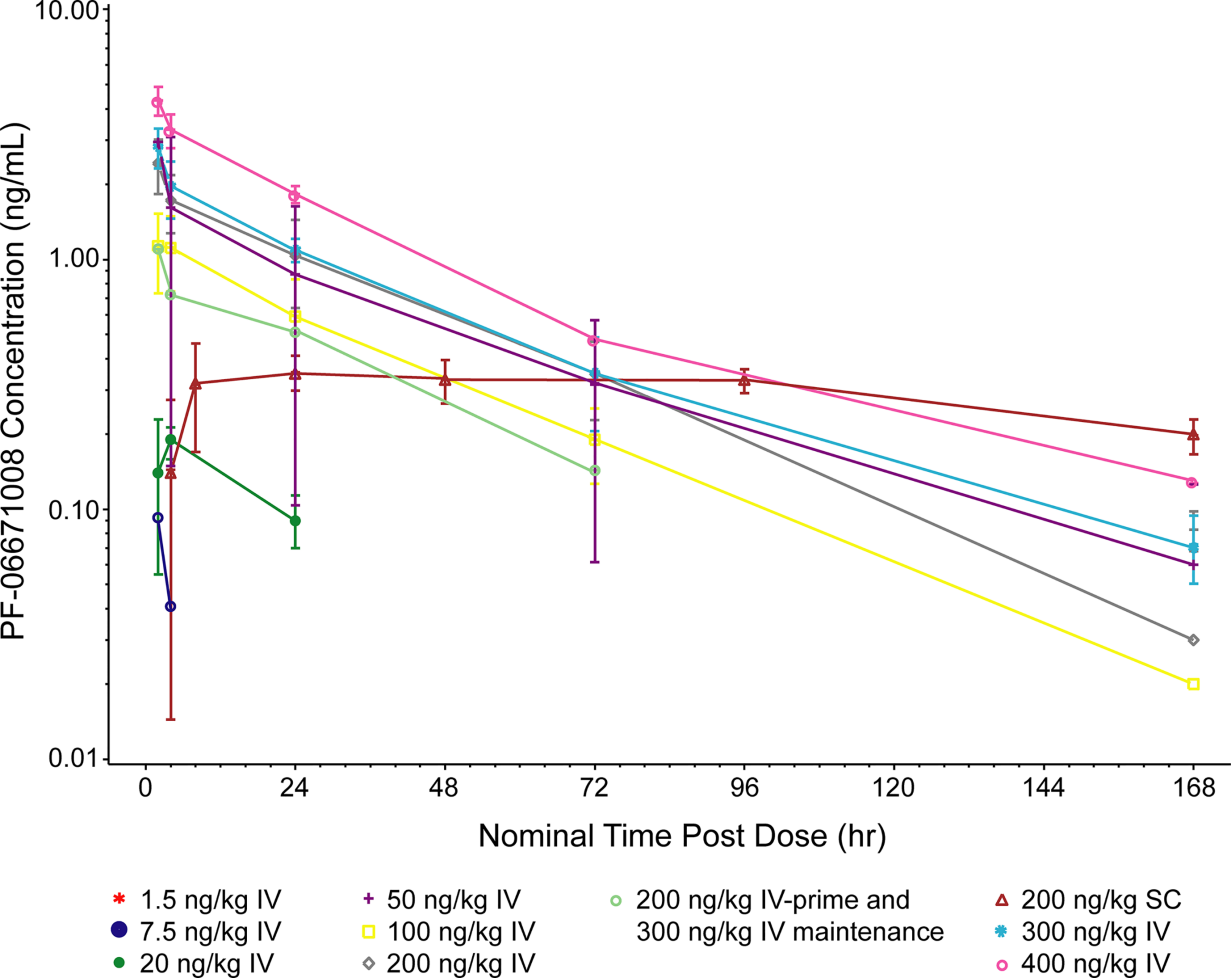
Pharmacokinetics of PF-06671008. 168 hr is equivalent to cycle 1 day 8, 0 hr. PF-06671008 was not detectable following the 1.5 ng/kg dose. The lower limit of quantification is 0.0500 ng/mL. Summary statistics were calculated by setting concentration values below the lower limit of quantification to zero. IV, intravenous; SC, subcutaneous.

Following IV dosing on cycle 1 day 1, the serum concentration of PF-06671008 appeared to decline in a biphasic manner at higher doses (50, 200, 300, and 400 ng/kg), whereas a monophasic decline was observed at doses of 7.5, 20, and 100 ng/kg (**Figure 2**). The C_max_ for PF-06671008 generally occurred at or shortly after the end of the infusion, except for the 20 ng/kg IV dose. Based on AUC_tau_ and C_max_ values, systemic exposure appeared to increase in a dose-dependent manner across the IV-dose groups (**Table 3**). Following IV dosing on cycle 1 day 1, t_1/2_ values for the 50–400 ng/kg doses were between 26.6 and 45.8 hours (**Table 3**).

**Table 3:** Pharmacokinetics of serum PF-06671008 following a single IV or SC dose (cycle 1, day 1).

	Parameter summary statistics^a^ by PF-06671008 Dose Level, ng/kg									
	1.5 IV	7.5 IV	20 IV	50 IV	100 IV	200 IV	300 IV	400 IV	200 IV-prime and 300 maintenance IV	200 SC
*N*, *n* ^1^, *n* ^2^	1, 1, 0	2, 2, 0	3, 3, 0	2, 2, 2	4, 4, 3	5, 5, 3	4, 4, 2	3, 3, 2	1, 1, 1	2, 2, 0
AUC_inf_, ng·hr/mL	NC	NC	NC	27.2, 123^b^	47.09 (41)	90.35 (19)	78.7, 97.0^b^	119, 189^b^	32.9^b^	NC
AUC_tau_, ng·hr/mL	0^b,c^	0, 1.06^b,c^	5.252 (25)	27.6, 116^b^	42.75 (35)	87.31 (29)	74.2, 89.1^b,d^	117, 177^b,d^	34.1^b^	47.5, 49.1^b^
C_max_, ng/mL	0^b,c^	0, 0.0932^b,c^	0.1926 (20)	0.675, 2.94^b^	1.108 (32)	2.008 (33)	2.387 (36)	4.049 (14)	1.10^b^	0.353, 0.418^b^
T_max_, hr	NC	2.02^b^	4.05 (2.00–4.08)	1.92, 2.65^b^	2.07 (2.00–4.10)	2.33 (2.00–3.98)	2.17 (2.05-3.63)	2.05 (2.05–3.00)	1.92^b^	8.12, 92.9^b^
t_1/2_, hr	NC	NC	NC	31.8, 41.1^b^	31.77 ± 7.6055	35.30 ± 5.4065	42.4, 45.8^b^	26.6, 40.4^b^	27.3^b^	NC
CL, mL/hr/kg	NC	NC	NC	0.416, 1.85^b^	2.191 (47)	2.236 (20)	3.07, 3.81^b^	2.19, 3.42^b^	6.08^b^	NA
V_ss_, mL/kg	NC	NC	NC	22.4, 85.2^b^	94.42 (36)	102.7 (27)	196, 199^b^	125, 132^b^	235^b^	NA

N, number of patients in the treatment group; n^1^, number of patients contributing to summary statistics for AUC_tau_, C_max_ and T_max_; n^2^, number of patients for t_½_, AUC_inf_, V_ss_, and CL.

a Geometric mean (geometric %CV) for all except: median (range) for T_max_; arithmetic mean ± standard deviation for t_½_.

b Individual value(s) reported for fewer than three patients.

c Patients with PK concentrations <LLOQ throughout their concentration-time profile, PK parameter AUC_tau_, and/or C_max_ = 0.

d n^1^, Two patients for AUC_tau_.

AUC_inf_, area under the serum concentration–time curve from time zero extrapolated to infinity; CL, clearance; CV, coefficient of variation; IV, intravenous; LLOQ, lower limit of quantification; NA, not applicable; NC, not calculated; SC, subcutaneous; t_1/2_, terminal half-life; V_ss_, steady state volume of distribution.

Following SC dosing (200 ng/kg) in two patients, C_max_ was observed at ~8 hours postdose in one patient and 93 hours postdose in the other patient. Serum concentrations of PF-06671008 gradually declined before administration of the next SC dose. C_max_ following SC dosing was lower than for the equivalent dose delivered by IV (**Table 3**).

At baseline, no patients had positive ADAs against PF-06671008. ADAs were not detected in any of the 25 patients who received IV administration of PF-06671008, regardless of IV dose level. Treatment-induced ADAs were detected in one of the two patients who received PF-06671008 at 200 ng/kg SC.

### Clinical Activity

**Figure 3:**
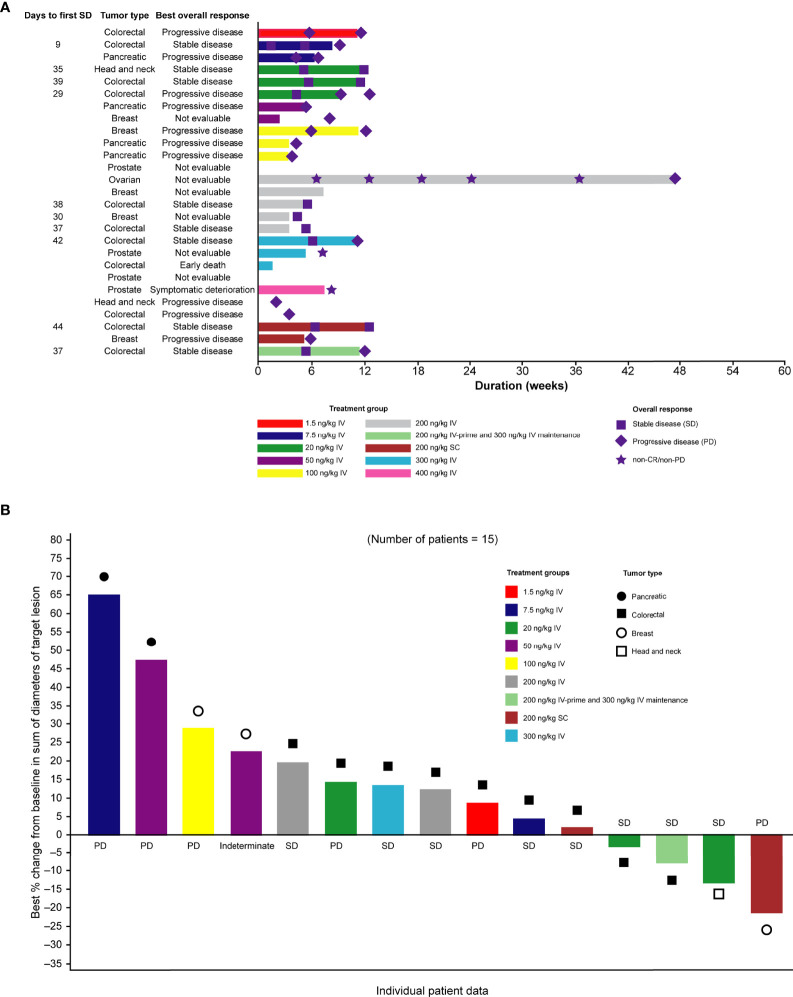
**(A)** Swimmer plot of duration of treatment, according to dose group and **(B)** waterfall plot of best percentage change in tumor size from baseline. Only patients who were assessed at day ≥35 after first dose are shown. CR, complete response; IV, intravenous; PD, progressive disease; SC, subcutaneous; SD, stable disease.

Of the 27 patients enrolled in the study, no patient achieved a confirmed complete or partial response and eight had stable disease, 10 had objective progression, one experienced symptomatic deterioration, and one had an early death. The best overall response was not determined for seven patients. In all, two patients remained on the study for at least 12 weeks and one patient remained on the study for at least 24 weeks (**Figure 3A**). Although the criteria for a partial response were not met based on RECIST v1.1, tumor shrinkage (a decrease of between 3% and 21% from baseline in overall diameter) was observed in four patients (**Figure 3B**).

### Pharmacodynamics

**Figure 4:**
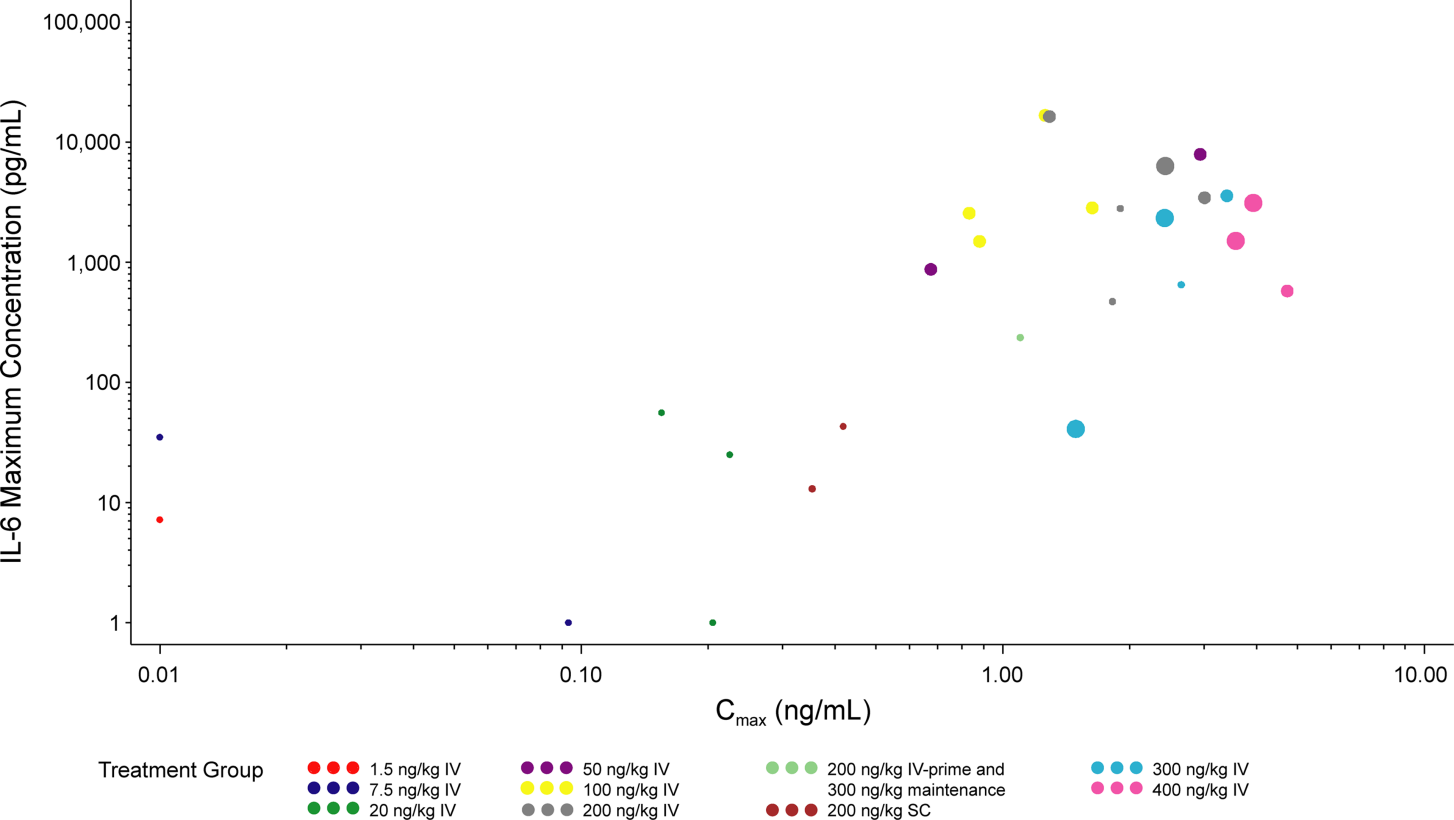
Maximum IL-6 concentration vs C_max_ (cycle 1). The LLOQ values were set to 0.01 ng/mL for C_max_ and 1 pg/mL for IL-6. Values >7900 for IL-6 were set to 7900 pg/mL. The size of the circles reflect the grade of CRS observed. Time frame for maximum IL-6 level, C_max_, and maximum CRS grade are both the first dosing interval during cycle 1 (time 0 hr to 168 hr after cycle 1 day 1 dose). C_max_, maximum serum concentration; CRS, cytokine release syndrome; IL, interleukin; IV, intravenous; SC, subcutaneous.

A dose-dependent increase in serum cytokine concentration was observed up to the 100 ng/kg dose. At doses above 200 ng/kg IV, observed CRS tended to be of a higher maximum grade (**Supplemental Figures 1A, B**). Serum cytokine concentrations appeared to be positively correlated with CRS grade (**Supplemental Figures 1A, B**). The Pearson correlation coefficients between serum cytokine concentration and CRS grade were 0.38 for IL-6 and 0.43 for IFN-γ. Serum cytokine peak concentration appeared to be positively correlated with C_max_, as shown for IL-6 in **Figure 4**. An increase in CRS grade was also observed with increasing C_max_, particularly for the 200, 300, and 400 ng/kg IV-dose groups (**Figure 4**). For most patients, serum cytokine levels were highest between 1 and 8 hours postdose in cycle 1 day 1, with a substantially smaller peak observed between 1 and 8 hours postdose in cycle 1 day 8 (**Supplementary Figure 2**). Based on the small number of patients in this study, the SC-dosing route appeared to generally induce less serum cytokine production and lower grade CRS than the IV-dosing route, when comparing similar nominal doses. However, the small number of patients involved make it difficult to draw meaningful conclusions. Nonetheless, these are hypothesis generating clinical findings that warrant further investigation.

## Discussion

Based on encouraging preclinical data, we conducted this first-in-human clinical study assessing the operating characteristics of PF-06671008, a novel T-cell–redirecting bispecific molecule targeting P-cadherin in patients with solid tumors. Patients received PF-06671008 at doses ranging from 1.5 ng/kg to 400 ng/kg across 10 dose-escalation and dose-regimen groups. The on-target toxicity of CRS was the most common TRAE. Although all cases of CRS resolved with supportive measures, concerns regarding the tolerability of the IV route of administration prompted an evaluation of both an IV-prime dose and SC-dosing strategy to potentially mitigate CRS. Unfortunately, the limited sample size of the IV-prime and the SC dosing cohorts limits our ability to make a definitive conclusion regarding these migration strategies. That said, our limited and preliminary data suggest less toxicity and should be considered hypothesis generating. Given the limited antitumor activity observed and the interest in exploring other potential targets with the T-cell–recruiting bispecific approach, the MTD and RP2D of PF-06671008 were not determined for this study.

CRS and its associated morbidity represent a clear potential limitation to T-cell–engaging therapies, and PF-06671008 exhibited a high proportion of this on-target AE; 78% of patients experienced CRS, with 19% of patients discontinuing the trial due to this event. Other notable AEs included transient lymphopenia, possibly representing pharmacologically related, transient redistribution of lymphocytes in response to treatment, as well as hypophosphatemia and gastrointestinal toxicities. Prior studies suggest that the unwanted immunologic effects of CRS may be mitigated by the use a priming dose (17). Topp et al. reported that the optimal blinatumomab dosing schedule was stepwise dosing with a lower dose followed by a higher target dose (17). Solitomab, a BiTE targeting EpCAM, exhibited significant AEs, which prevented dose escalation to therapeutic levels (19). Interestingly, CRS was less pronounced with a low-dose run-in treatment schedule (19). Our observations from this study (albeit with a limited dataset) also suggest more studies should explore the implementation of a priming dose to reduce CRS.

A key innovation of our study was the careful evaluation of PK relative to cytokine profiles and CRS presentation. Our data indicate that the C_max_ of PF-06671008 correlates with CRS; thus, it stands to reason that modification of drug exposure might decrease the incidence of these deleterious AEs associated with T-cell–engaging therapy. The implementation of SC dosing may represent another potential strategy to mitigate CRS for this class of molecule. As only two patients received SC dosing, firm conclusions cannot be made, though it is interesting to observe qualitatively changes to the dose-exposure relationship with the SC approach. At the same dose as IV administration, SC dosing resulted in a much lower (~five-fold lower) C_max,_ and a marginally lower (~two-fold lower) AUC. The difference in C_max_ was considerably larger, and appeared to translate to lower cytokine release, even when compared with the IV cohort at a lower dose level (e.g., 100 ng/kg) with a similar AUC. Indeed, careful analysis suggests that C_max_ of PF-06671008 correlates with maximum cytokine level; pharmacokinetic methods that alter the route of administration to “flatten” the exposure curve may reduce CRS and may yield more tolerable drug delivery. Further investigation to refute or accept this provocative hypothesis is warranted and will require a greater number of patients dosed SC.

The findings described in this article are also useful for the selection of future monoclonal antibody targets. Preclinical data demonstrated that PF-06671008 resulted in tumoral shrinkage, and P-cadherin has been identified as a TAA in certain gastrointestinal cancers. However, the lack of major clinical objective responses, despite favorable PK and on-target AEs, requires a reevaluation of the suitability of P-cadherin as a drug target in advanced solid tumors, at least with the current strategy (10, 14). It is well established that P-cadherin’s role in oncogenesis is context dependent — strongly expressed in well-differentiated tumors but downregulated in poorly differentiated tumors (20). Furthermore, P-cadherin knockdown in a colon cancer cell line induces cell dissociation, migration, and invasion (20). Thus, P-cadherin may be less relevant in advanced metastatic disease. Indeed, other monoclonal antibodies targeting P-cadherin have shown lackluster efficacy, with low objective response rates. This is reinforced by the findings from a phase 1 study of an Yttrium-90-conjugated, P-cadherin–targeted chimeric monoclonal antibody for the treatment of advanced solid tumors, in which a single complete response and no partial responses were observed (21). Enrichment strategies and prescreening for P-cadherin high tumors (not required in the phase 1 dose-escalation portion of this study) may also be needed for observation of antitumor activity in treatments targeting P-cadherin. Alternatively, issues specific to solid tumors might limit antitumor activity; these issues include inherent tumor heterogeneity related to P-cadherin expression, as well as immunosuppressive stromal cells and the tumor microenvironment blunting an adequate CD8+ T-cell response (22). Essentially, the complexities of the human solid tumor immune microenviroment may have not been fully capitulated in the preclinical models and thus led to the discordance in the preclinical and clinical data.

PF-06671008 was one of the first molecules in this structural class targeting solid tumors, and the associated immunologic effects as well as on-target toxicity support the pharmacodynamic activity of the molecule. A recent phase 1 study showed that pasotuxizumab, a BiTE targeting the prostate-specific membrane antigen, exhibited preliminary efficacy in patients with advanced castration-resistant prostate cancer (23). The novel carcinoembryonic antigen T-cell bispecific (CEA-CD3 TCB) antibody (RG7802, RO6958688) showed antitumor activity in patients with advanced colorectal cancer, which was enhanced in combination with atezolizumab (24). Taken together, these previous reports and the data from this study support the utility of this class of molecules for the treatment of solid tumors.

This study has several strengths and notable limitations. The application of DART technology created a molecule with favorable PK properties and preclinical stability, which allowed effective translation to the clinic and obviated the need for continuous IV dosing. This study, through the evaluation of multiple schedules, also provides a hypothesis for the potential value of a priming dose and, importantly, SC administration in reducing the intensity of CRS. Shortcomings of the study include the small number of patients with each tumor type, making it difficult to evaluate the antitumor activity of PF-06671008, as well as the lack of *a priori* determination of P-cadherin levels in patients’ tumors. The prolonged dose-escalation phase, due to the low MABEL-derived starting dose, is a known challenge with developing CD3 bispecific molecules (25, 26).

Based on the totality of the data, the study was terminated given the lack of activity of PF-06671008. Owing to the early termination, part 2 of the study (dose expansion) was not conducted and no further development is planned. Although further development of PF-06671008 has been halted, the findings from this study provide valuable insights into optimal dosing and management of AEs for this class of molecule, which should inform future efforts to develop more effective therapies against solid tumors.

## Data Availability Statement

The raw data supporting the conclusions of this article will be made available by the authors, without undue reservation.

## Ethics Statement

The studies involving human participants were reviewed and approved by institutional review boards at MD Anderson Cancer Center, The University of Utah, Memorial Sloan Kettering, and University Hospitals. The patients/participants provided their written informed consent to participate in this study.

## Author Contributions

SM, DH, JH, and MS conceived the study. XC, SM, AH, DH, CK, JH, AM, MS, CB, and GW designed the study. XC, IG-L, AD, DH, JH, MS, CB, and AF were involved in data collection. XC, AD, and CB conducted the statistical analysis. All authors participated in the interpretation/analysis of study results, and in the drafting, critical revision, and approval of the final version of the manuscript.

## Funding

This study was sponsored by Pfizer. Pfizer was involved in the study design, the collection, analysis and interpretation of the data, and the preparation of the manuscript. The authors had final authority, including on the choice of journal, on all aspects of the manuscript content and development.

## Conflict of Interest

JH, grant support from Bristol Myers Squibb and consultancy fees from Bristol Myers Squibb, Merck, Eisai, Exelexis, Eli Lilly, QED, Adaptimmune, Imvax, and Cytomx. IG-L, consultancy fees from Eisai and Array, research support (institution) from Amgen, Bridgebio, Jacobio, Tolero, Trishula, Bayer, Seattle Genetics, Lilly, Incyte, GSK, Pfizer, and Redhill. AD, fees for advisory boards from AstraZeneca, Seattle Genetics, Ipsen, Bayer, Bristol Myers Squibb, Merck and G1 Therapeutics. DH, research/grant funding (institution) from AbbVie, Adaptimmune, Adlai-Nortye, Amgen, AstraZeneca, Bayer, Bristol Myers Squibb, Daiichi-Sankyo, Deciphera, Eisai, Erasca, Fate Therapeutics, Genentech, Genmab, Ignyta, Infinity, Kite, Kyowa Kirin, Lilly, LOXO, Merck, Medimmune, Mirati, Mologen, Navier, NCI-CTEP, Novartis, Numab, Pfizer, Pyramid Bio, SeaGen, Takeda, Teckro, Turning Point Therapeutics, Verstatem, and VM Oncology; travel/accommodation/expenses from Bayer, Genmab, AACR, ASCO, SITC, and Telperian; consulting/speaker/advisory role for Adaptimmune, Alpha Insights, Acuta, Alkermes, Amgen, Aumbiosciences, Axiom, Baxter, Bayer, Boxer Capital, BridgeBio, COR2ed, COG, Ecor1, Genentech, Gilead, GLG, Group H, Guidepoint, HCW Precision, Immunogen, Infinity, Janssen, Liberium, Medscape, Numab, Oncologia Brasil, Pfizer, Pharma Intelligence, POET Congress, Prime Oncology, Seattle Genetics, ST Cube, Takeda, Tavistock, Trieza Therapeutics, Turning Point, WebMD, and Ziopharm; other ownership interest in Molecular Match (Advisor), OncoResponse (Founder), Telperian (Advisor). AM and CB, employed by Pfizer and hold stock in Pfizer. GW, AH, AF, SM, CK, XC, and MS, previously employed by Pfizer and hold stock in Pfizer.

## Publisher’s Note

All claims expressed in this article are solely those of the authors and do not necessarily represent those of their affiliated organizations, or those of the publisher, the editors and the reviewers. Any product that may be evaluated in this article, or claim that may be made by its manufacturer, is not guaranteed or endorsed by the publisher.

## Abbreviations

ADA, antidrug antibodies; AE, adverse event; AUC, area under the serum concentration–time curve; BiTE, bispecific T-cell engager; C_max_, maximum serum concentration; *CDH1*, E-cadherin; *CDH2*, N-cadherin; *CDH3*, P-cadherin; CRS, cytokine release syndrome; DART, Dual Affinity Re-Targeting Platform; DLT, dose-limiting toxicity; IFN, interferon; IL, interleukin; IV, intravenous; MABEL, minimal anticipated biological effect level; MTD, maximum tolerated dose; PK, pharmacokinetics; RECIST, Response Evaluation Criteria in Solid Tumors; RP2D, recommended phase II dose; SC, subcutaneous; TAA, tumor-associated antigens; t_1/2_, terminal half-life; T_max_, time to maximum serum concentration; TNF, tumor necrosis factor; TRAE, treatment-related adverse event; VH, very heavy chain; VL,very light chain.
