# Supplementary figures and images for: A Phase 1 Dose-Escalation Study of PF-06671008, a Bispecific T-Cell-Engaging Therapy Targeting P-Cadherin in Patients With Advanced Solid Tumors
Source: Front Immunol. 2022 Apr 14;13:845417. doi: 10.3389/fimmu.2022.845417 (PMC9047766; doi:10.3389/fimmu.2022.845417)

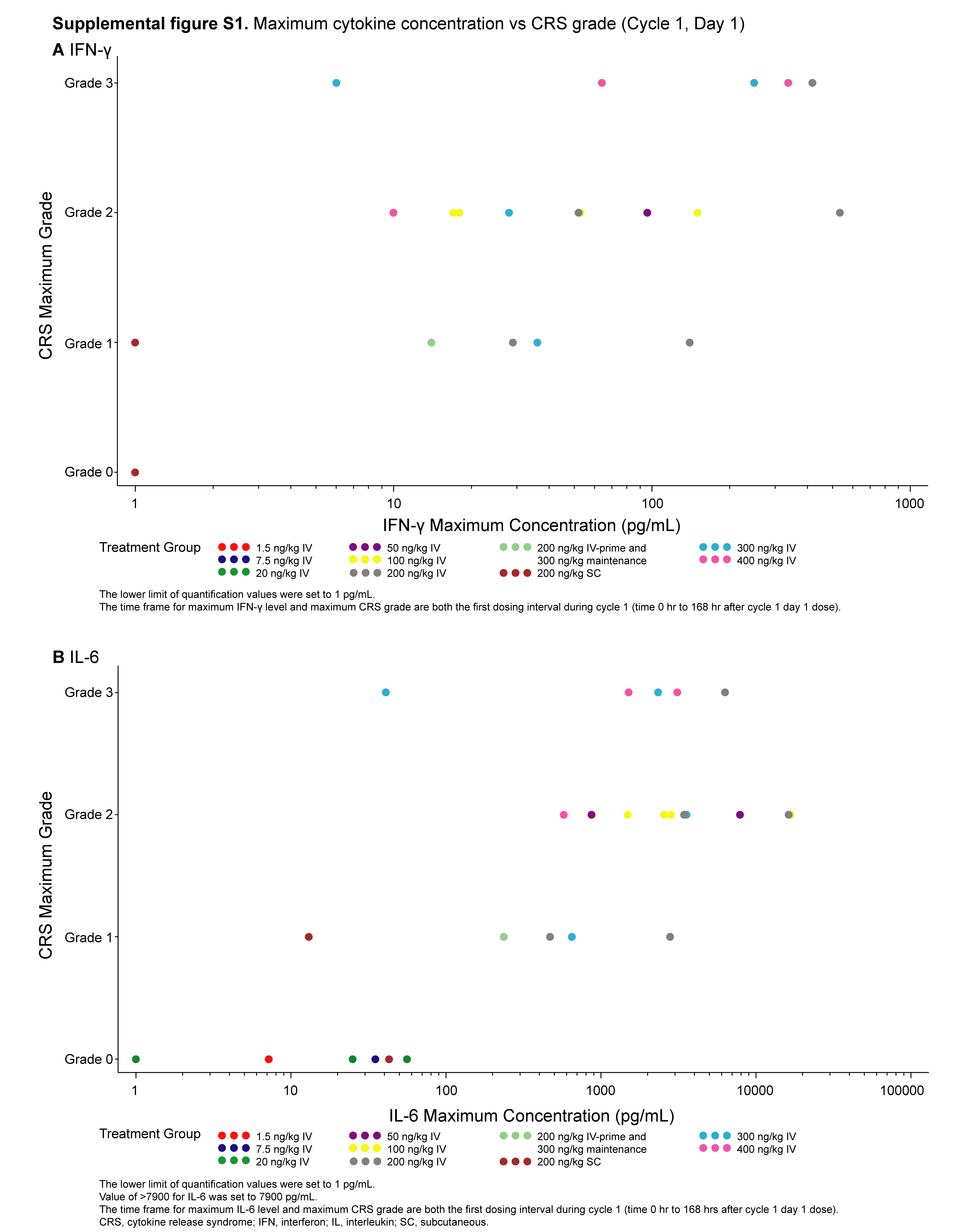

Supplement: Supplementary file 1 [file Image_1.tif]

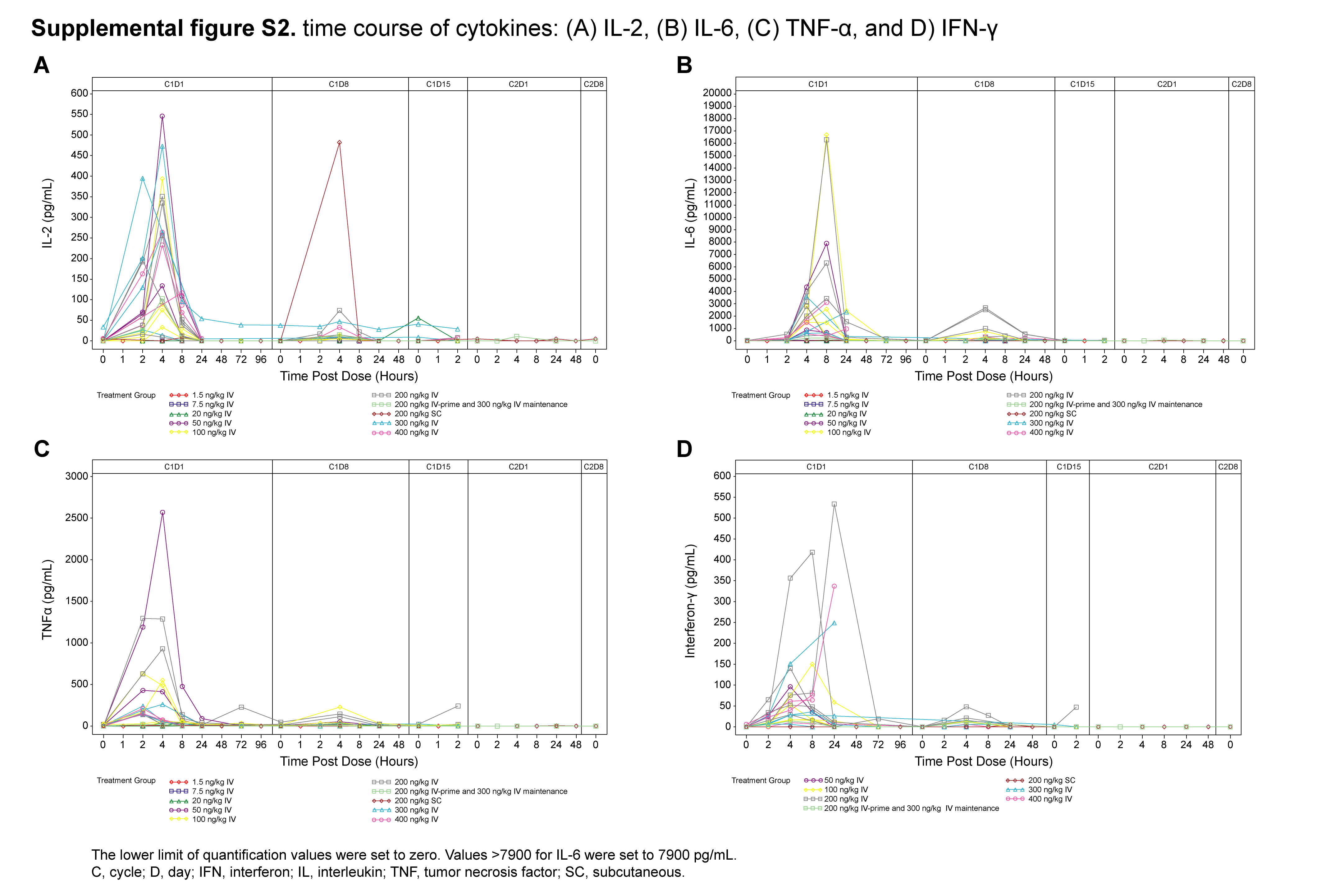

Supplement: Supplementary file 2 [file Image_2.tif]
